# Towards new approach methodologies for biological therapeutics: a novel model-informed metric to assess immunogenicity risk

**DOI:** 10.3389/fimmu.2025.1677925

**Published:** 2025-11-03

**Authors:** Rachel H. Rose, Aban Shuaib, Manon Wigbers, Maryam Khalifa, Andrzej M. Kierzek, Piet H. van der Graaf

**Affiliations:** ^1^ Certara Predictive Technologies, Applied BioSimulation, Sheffield, United Kingdom; ^2^ School of Biosciences, Faculty of Health and Medical Sciences, University of Surrey, Guildford, United Kingdom; ^3^ Leiden Academic Centre for Drug Research, Leiden, Netherlands; ^4^ Cincinnati Children’s Hospital Medical Center, Cincinnati, OH, United States

**Keywords:** immunogenicity, anti-drug antibody, biotherapeutic, quantitative systems pharmacology, pharmacokinetics, model-informed drug development

## Abstract

Immunogenicity poses a significant challenge in biotherapeutics development due to the formation of anti-drug antibodies (ADA), which can alter drug pharmacokinetics (PK) and reduce efficacy. However, ADA presence does not always correlate with a clinically relevant reduction in efficacy, or in some cases can be managed by adjusting dosing regimens. Current preclinical strategies focus on predicting the propensity for ADA development, but do not assess the liability for ADA to impact PK. Quantitative systems pharmacology (QSP) models integrate knowledge of biological mechanisms with physiological and drug-specific parameters to predict ADA dynamics and their effect on PK. This study describes recent progress in using QSP models to predict the incidence of immunogenicity and the impact of ADA on PK. We report continued challenges in accurately predicting ADA incidence from available data from experimental and computational methods used in immunogenicity risk assessment. However, across 13 monoclonal antibodies and fusion proteins, the model accurately predicted ADA impact on drug concentration in ten cases, Furthermore, the ADA to drug concentration ratio was identified as a strong predictor of clinically relevant immunogenicity and drug exposure impact.

## 1 Introduction

The number of biotherapeutics in development and approved by regulators has sharply increased over the last three decades, with biologics license applications making up almost 30% of FDA approved drugs over the last 10 years (1). While these therapies have provided significant benefits to patients, challenges remain in understanding and mitigating the development of unwanted immunogenicity to protein biotherapeutics. Immunogenicity occurs when there is activation of a humoral immune response that results in development of anti-drug antibodies (ADAs) that can enhance clearance of the biotherapeutic, reduce efficacy or evoke safety issues that may limit the population that benefits from treatment or result in clinical stage failure of a development program. However, ADA positivity is not always associated with loss of drug exposure, efficacy or adverse safety outcomes, suggesting a lack of clinical relevance for some drugs. Thus, in pre-clinical and early clinical immunogenicity assessment, it is desirable to predict and measure not just the incidence of immunogenicity, but whether ADA will be clinically relevant. The US Food and Drug Administration (FDA) recently announced plans to take steps towards phasing out requirements for animal testing for antibodies and other drugs in favour of more predictive *in silico* and *in vitro* human-relevant methods (2). While this announcement was driven by multiple important scientific, ethical and cost considerations, the poor predictivity of animal models for multiple applications including immunogenicity was cited as driving the need for alternative, innovative new approach methodologies.

Multiple factors have been implicated in the development of immunogenicity to biotherapeutic, including product-related factors (e.g. protein sequence, host cell impurities, excipients, aggregation), patient-related factors (e.g. genetics, disease status, age) and trial design factors such as dose amount and frequency, route of administration or co-medication (3, 4). Current pre-clinical immunogenicity risk assessment approaches for therapeutic proteins frequently include *in silico* and *in vitro* methods that evaluate risk factors at different stages of the immune response to characterise some of these factors. *In silico* approaches are well established in the assessment of protein sequence similarity to the germline and Major Histocompatibility Complex class II (MHCII) binding and antigen presentation (5, 6). *In vitro* approaches include dendritic cell uptake and activation assays, MHC associated peptide proteomics to measure presented antigens and T cell activation and proliferation assays. Prediction of B cell epitopes and B cell activation remain more challenging and less well established [reviewed in (5, 7)]. However, integrating these multiple outcomes to assess the risk and clinical relevance of immunogenicity remains a challenge.

Quantitative systems pharmacology (QSP) and physiologically-based pharmacokinetic (PBPK) models are widely used in drug development and regulatory approval to inform decision making, contributing to reduced development timelines and costs (8–10). These mechanistic computational models integrate drug-specific data from multiple sources including *in silico* predictions*, in vitro* and *in vivo* experiments with knowledge of physiological and pathophysiological processes to understand how drugs and biological systems interact to determine concentrations, pharmacological activity and toxicity.

The Immunogenicity Simulator (IG Simulator), a QSP platform model, has been developed with the goal of predicting and managing clinical immunogenicity to non-self therapeutic proteins (11). The IG Simulator integrates mathematical models of immunology with a PBPK model and physiological parameters and drug-specific parameters derived from experimental and computational approaches. The approach builds on efforts in the mechanistic modelling of immunogenicity, most notably by Chen et al. (12, 13), and PBPK modelling of therapeutic proteins (14). Previously we have presented an evaluation of the application of the IG Simulator to predict immunogenicity of 10 monoclonal antibodies as part of the learn and confirm approach to model development (15). One of the challenges identified in this work was the non-trivial nature of comparing predicted and observed exposure loss in ADA positive subjects. In the current analysis we evaluate the performance of the IG Simulator in predicting whether there is a significant impact of ADA positivity on PK versus reported outcomes in clinical studies. Furthermore, we describe a novel interpretation of model outcomes using the model derived metric of ADA:drug concentration ratio, which is shown to be highly predictive of the likelihood of clinically relevant immunogenicity with an impact on drug exposure.

## 2 Materials and methods

### 2.1 Model description

The IG Simulator V7 (Certara Predictive Technologies, Sheffield, UK) is a QSP model which integrates mechanistic, ordinary differential equation models of the immune response and drug PK, connected via the concentrations of drug in model compartments (11, 15). The starting point for the IG Simulator was the work of Chen, Hickling, and Vicini (CHV) (12, 13), who developed a multiscale model of biological processes involved in the humoral immune response to an antigen. The CHV model mechanistically represents the subcellular level processes of antigen presentation by dendritic cells, a cellular level model of the kinetics of immune cells including the activation, differentiation and proliferation of drug-specific CD4+ T cells and B cells and binding ADA production by plasma cells and a PK model of the protein antigen. Several modifications were made to the CHV model. First the compartmental PK model for the drug was replaced with a minimal PBPK module comprised of plasma, lymph node, and a lumped tissue compartment that was further divided into vascular, endosomal, and interstitial compartments (14). The immune response model was compartmentalised to the lymph node, blood, and vascular compartment and the immune response driven by interaction with antigenic protein concentration with the biologically relevant compartment, as described previously (16). Antigen presentation prediction methods trained on mass spectrometry eluted ligand data in addition to binding affinity data have superior performance in the prediction of antigen presentation versus methods trained on binding affinity alone (17, 18). While binding affinity data specifically characterises the process of peptide binding to human leukocyte antigens (HLAs), eluted ligand data measures the processed and presented peptides bound to HLAs on antigen presenting cells, thus includes the contribution of multiple steps in the antigen presentation pathway. Elution rank is a relative score that allows cross-allele comparisons and evaluation of peptides across different class II HLAs (17). To enable input of predicted elution rank from NetMHCIIpan 4.0 (17), the subcellular model of antigen presentation from Chen et al. (13), was removed, and the T cell activation function (D_N_Epitope) modified to use elution rank (EL_rank) specific to a peptide and HLA allele combination expressed in an individual. Experimental measurements of the dendritic cell uptake of intact biotherapeutics were directly input as a scaling factor multiplying antigen concentration (DC_Uptake) to account for drug-specific uptake rates.


$$\begin{array}{c} D\_N\_Epitope=\frac{ID\_m}{\left(ID\_m+Ttot\right)}\\ \times \frac{DC\_Uptake\times AgVS\times \sum_{Allele}{\left(100/EL\_rank\_Epitope\_Allele-1\right)}^{2}}{DC\_Uptake\times AgVS\times \sum_{Allele}{\left(100/EL\_rank\_Epitope\_Allele-1\right)}^{2}+K\_Ag\_N} \end{array}$$

Where ID_m is the number of mature dendritic cells; Ttot is the total number of drug-specific T cells, summed for naïve, activated and memory T cells; AgVS is the drug concentration in vascular space (AgVS); and elution rank (EL_rank) for the specific peptide and allele and the experimentally derived antigen uptake rate by dendritic cells (DC_Uptake), and K_Ag_N is a constant defining half-maximal activation of T cells by presented antigen, calibrated to minimise the root mean square error between predicted and observed immunogenicity incidence for the 13 drugs in this study. Methotrexate co-medication has been associated with a decrease in ADA concentrations and incidence in clinical trials (19–21), and for several of the drugs under investigation methotrexate was co-administered in clinical studies reporting immunogenicity (**Supplementary Table 3**). The PK of oral methotrexate was captured by a two compartment PK model with first order absorption (22). The pharmacodynamics was modelled using an inhibitory E_max_ model to describe the inhibition of the T cell proliferation rate (ρ_AT_). An estimated IC_50_ of 283 nM captured the reduction in ADA incidence at high, medium and low methotrexate doses (20) (**Supplementary Figure 1**).

### 2.2 Compound-specific input parameters

Compound specific parameters for the PBPK model are summarised in **Supplementary Table 1**. Plasma clearance and bioavailability and absorption rate for subcutaneously administered drugs, were optimised to capture clinical data (**Supplementary Figure 2**) using the Nelder Mead method implemented in the parameter estimation toolbox in QSP Designer V2 (23).

Where available, T cell epitope selection was informed by eluted peptides from MHC-associated peptide proteomics (MAPPs) data (**Supplementary Table 2**). When MAPPs data was not available, the full primary protein sequence was analysed for risk of T cell epitopes. A list of all possible 15-mers derived from the eluted peptides or primary protein sequence were screened using BLAST (blast-2.14.0+) against the UniProt Knowledgebase human reference proteome (24) to identify and remove all sequences that align with an endogenous human protein assuming immune tolerance to peptides from self-proteins. Elution rank for alleles and haplotypes of the three classical class II HLAs, DR, DQ and DP from the Immune Epitope Database (IEDB) HLA reference set (25, 26) was predicted using elution rank reported by NetMHCIIpan (17). Up to five non-overlapping peptides with the strongest binding (lowest elution rank) across the HLA reference set were selected as the highest risk potential T cell epitopes in simulations. Including more than five potential T cell epitopes did not increase predicted ADA incidence for the test set (data not shown). Allele and haplotype frequencies were extracted from the allele frequencies net database (27). Where available, published experimental data for the initial fraction of drug-specific naïve T cells and the dendritic cell uptake rate were also integrated in the model (**Supplementary Table 2**).

### 2.3 Virtual clinical trial simulation

250 virtual subjects were generated to include physiological variability in compartment volumes and fluid flow rates, drug clearance and HLA genetics. Physiological parameter sets for the PBPK model, including volumes of plasma, lymph nodes and tissue compartments, blood and lymph flows, endogenous IgG concentration and FcRn concentrations were generated using the Simcyp Simulator V19 (Certara Predictive Technologies, Sheffield, UK) minimal PBPK model for monoclonal antibodies. Variability in drug clearance was generated by Monte Carlo sampling of the lognormal parameter distribution defined by the mean and coefficient of variation.

Variability in HLA genetics was considered for the three classic class II HLAs, DRB1, DP, and DQ. DRB1 allele and DP and DQ haplotype distributions for the North American and European populations were calculated using data available at the Allele Frequency Net Database (27). For all publications from studies performed in the population, allele or haplotype frequencies were averaged, weighting by study size. The analysis included all 11 DRB1 alleles, 6 DQ and 6 DP haplotypes included in the IEDB HLA Reference Set, representing the most common specificities in the general population (26). For each virtual subject, two alleles or haplotypes at each locus were randomly selected according to their frequencies.

Comparator clinical studies were selected as studies that report sufficient information to enable the study design to be reproduced, including sufficient information on the drug dosing regimen, sampling times for analysis of ADA and drug concentrations and assessment of the ADA incidence and the impact of ADA on PK. For simulations, drug dosing regimen, study duration, and sampling times for plasma drug and ADA concentration measurements were matched to the reported trial design for a comparator study (**Supplementary Table 3**). The QSP model predicts the temporal profiles of drug, ADA and immune complex concentrations for each virtual individual, which vary depending on the individual physiological, genetic, compound-specific and trial-specific parameters. A virtual subject was identified as ADA positive if their total ADA concentration exceeds the concentration threshold for ADA positivity at one or more sampling time for ADA assessment. The threshold for ADA positivity either used the value reported in the comparator clinical study, or was assumed to take the FDA minimum recommended sensitivity of 100 ng/mL if ADA threshold concentration was not reported (28) (**Supplementary Table 2**).

To assess the impact of ADA on drug PK, clinical studies compared drug concentrations for individuals classified as ADA positive and ADA negative at one or more sampling times at which both ADA and drug concentrations were measured. For most studies sampling times were pre-dose and correspond to drug trough concentrations. Exceptions are rituximab, for which only two doses were administered and frequent sampling of PK and ADA was used, and ustekinumab, for which sampling was both at the trough concentration and halfway through the dosing interval. Different studies used different statistical methods for evaluating the impact of ADA on PK, and the study authors’ conclusions are taken directly for comparison to simulation outcomes. For simulations, a consistent statistical approach was used. The impact of ADA on PK was assessed by comparing free drug concentration for subjects assigned as ADA positive and negative at the final concentration sampling time reported in the clinical study using a Wilcoxon rank sum test (p < 0.05).

To assess relationship between drug concentration, ADA concentration and the impact of ADA on PK, PK only simulations were run for the same virtual subjects (i.e. the same parameter set) by deactivating the immune system model, leaving only the PBPK model active. This PK only simulation assessed drug concentration for all virtual subjects in the absence of ADA. The molar concentration ratio of ADA to drug in the absence of ADA ([ADA]:[Drug]) was calculated for each virtual subject for the maximum total ADA concentration versus the drug concentration at the final ADA sampling time in the clinical study.

All simulations were performed using QSP Designer V2 and *post hoc* analysis and plotting were performed in MATLAB R2024b.

## 3 Results

**Figure 1:**
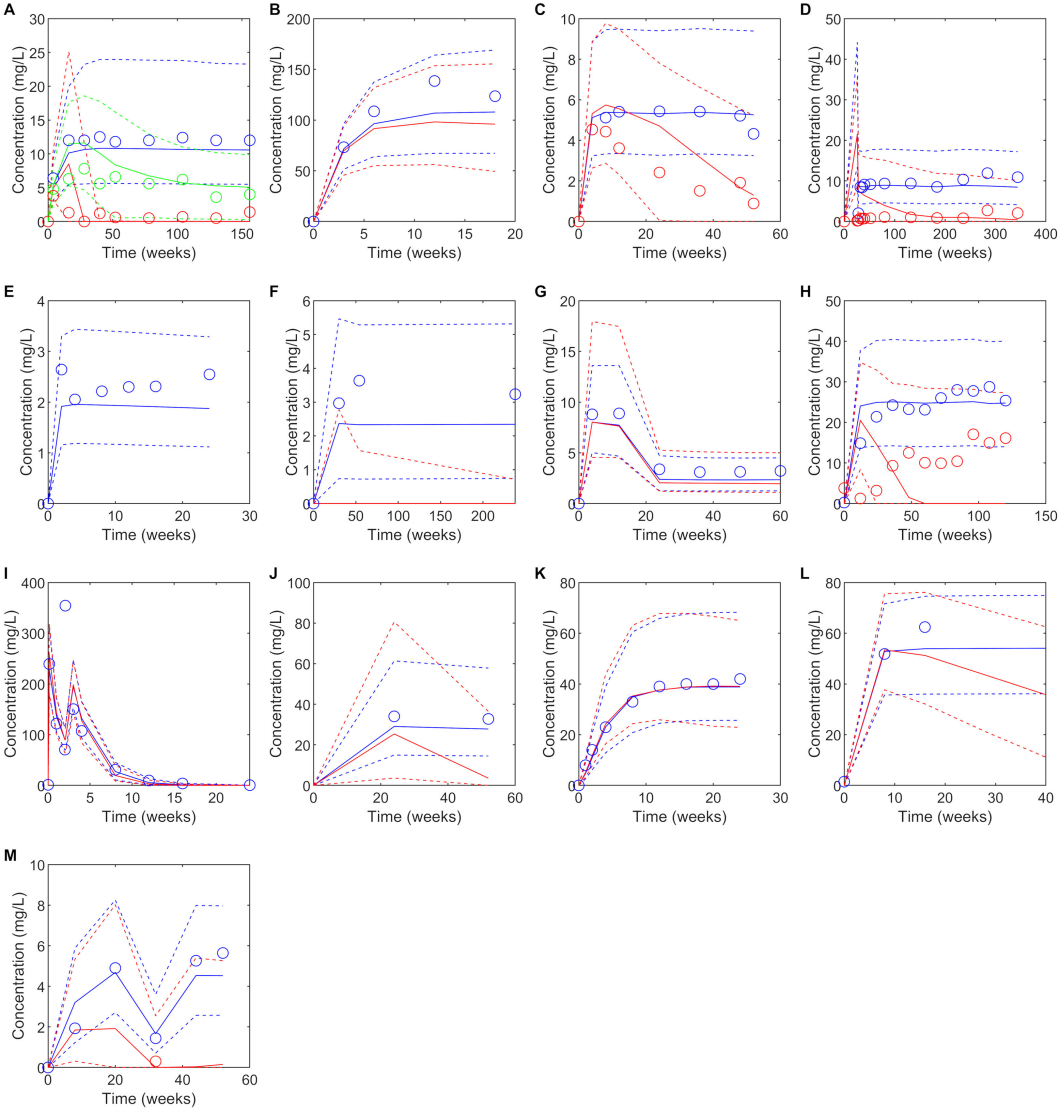
Predicted versus observed plasma concentrations at reported PK sampling times for ADA positive (red), low titer ADA positive (green, adalimumab only) and ADA negative (blue) subjects. Solid lines are the median of the simulated virtual populations are dashed lines are the 5^th^ and 95^th^ percentile, open circles are the reported clinical data. **(A)** 40 mg SC adalimumab Q2W **(B)** 15 mg/kg bevacizumab Q3W for 6 doses **(C)** 150 mg SC bococizumab Q2W **(D)** 400 mg SC certolizumab pegol Q2W for three doses followed by 200 mg SC certolizumab pegol Q2W (45), **(E)** 50 mg SC etanercept Q1W **(F)** 3 mg/kg IV infliximab on weeks 0,2 and 6 then Q8W **(G)** initial dose 160 mg SC ixekizumab, followed by 80 mg Q2W to week 12 then 80 mg Q4W to week 60, **(H)** 300 mg IV natalizumab Q4W for 120 weeks **(I)**1000 mg IV rituximab on weeks 0 and 2, **(J)** 300 mg SC secukinumab Q1W for 5 weeks, then Q4W **(K)** 162 mg SC tocilizumab Q1W for 24 weeks (65) **(L)** Initially 4 mg/kg IV trastuzumab, followed by 2 mg/kg Q1W **(M)** Initially 130 mg IV ustekinumab, then 90 mg Q8W. Source of observed data is defined in **Table 1**.

The developed PBPK models for each drug in the absence of ADA adequately captured single dose and multiple dose plasma concentration profiles for the 13 drugs (**Figure 1**, **Supplementary Figure 2**).

Predicted immunogenicity incidence was compared to the reported ADA incidence for the benchmark study for which the virtual study design was matched, as well as the range of ADA incidence reported for the monoclonal antibodies across multiple studies. When comparing to the outcome reported for the benchmark study, a trend was observed toward overprediction of the incidence of immunogenicity reported in the comparator study to which the virtual clinical trial design was matched. While 4/6 drugs with moderate to high immunogenicity (defined as >10% ADA incidence (29)) were correctly predicted having a moderate to high incidence of ADA positivity, only 2/7 drugs with low immunogenicity (defined as ≤10% ADA incidence) were correctly predicted as having low immunogenicity while for 5/7 drugs immunogenicity was overpredicted (**Table 1**). We note that considerable variability in ADA incidence was reported across different clinical trials for the same drug (**Table 1**).

**Table 1:** Summary of predicted versus observed ADA incidence and impact of ADA on PK.

Drug	ADA+ incidence (%)		Impact of ADA positivity on plasma concentration		Reference
	Observed (comparator study [range])	Predicted	Observed	Predicted	
Adalimumab	50 [0-95.9]	3.2	↓	↓	(20, 30, 38, 39)
Bevacizumab	2.5 [0-16.1]	37.2	→	→	(40–42)
Bococizumab	48 [7-50.3]	40.4	↓	↓	(31, 43, 44)
Certolizumab pegol	25.3 [3.1-37]	73.2	↓	↓	(45–47)
Etanercept	6.9 [0-14.1]	0	→	NA	(48–50)
Infliximab	35 [5.1-61]	14.8	↓	↓	(51–53)
Ixekizumab	17.4 [5-17.4]	4.4	→	→	(54, 55)
Natalizumab	9.1 [3-92]	43.2	↓	↓	(56–58)
Rituximab	23 [5.5-37]	18	↓	↓	(59–62)
Secukinumab	0–1 [0-2.3]	4.8	→	↓	(63, 64)
Tocilizumab	0.8 [0-91.9]	12.4	→	→	(65–67)
Trastuzumab	0.3 [0-10]	44.8	→	↓	(68–70)
Ustekinumab	2.3 [2.3-48.2]	30	↓	↓	(71, 72)

NA, not applicable, no ADA positive individuals predicted. Down arrows represent a decrease in drug concentration or increase in clearance for ADA positive versus ADA negative individuals, horizonal arrows represent no significant impact on drug concentration reported/predicted.

Despite the discrepancy in predicted versus observed ADA incidence, there were only two drugs, secukinumab and trastuzumab, for which the model predicted a significant reduction in plasma concentration in ADA positive subjects that was not seen in the clinical studies and in both cases the number of ADA positive subjects in the clinical study was very small (<1%) (**Table 1**, **Figure 1**). For 10/13 drugs the model correctly predicted whether ADA would be associated with a significant reduction in plasma concentration as observed in the clinical study. It was notable that for three drugs, bevacizumab, ixekizumab, and tocilizumab, despite overprediction of ADA incidence, ADA positivity was not associated with a reduction in plasma concentration, in agreement with clinical observations (**Table 1**). For etanercept, the model predicted no ADA positive subjects, so the impact of ADA on the drug concentration could not be assessed. For drugs in which ADA was associated with a reduction in drug concentration, considerable variability in the drug concentration in ADA positive individuals was predicted, with drug concentration remaining within the range of those for ADA negative concentrations in some cases (**Figure 1**).

**Figure 2:**
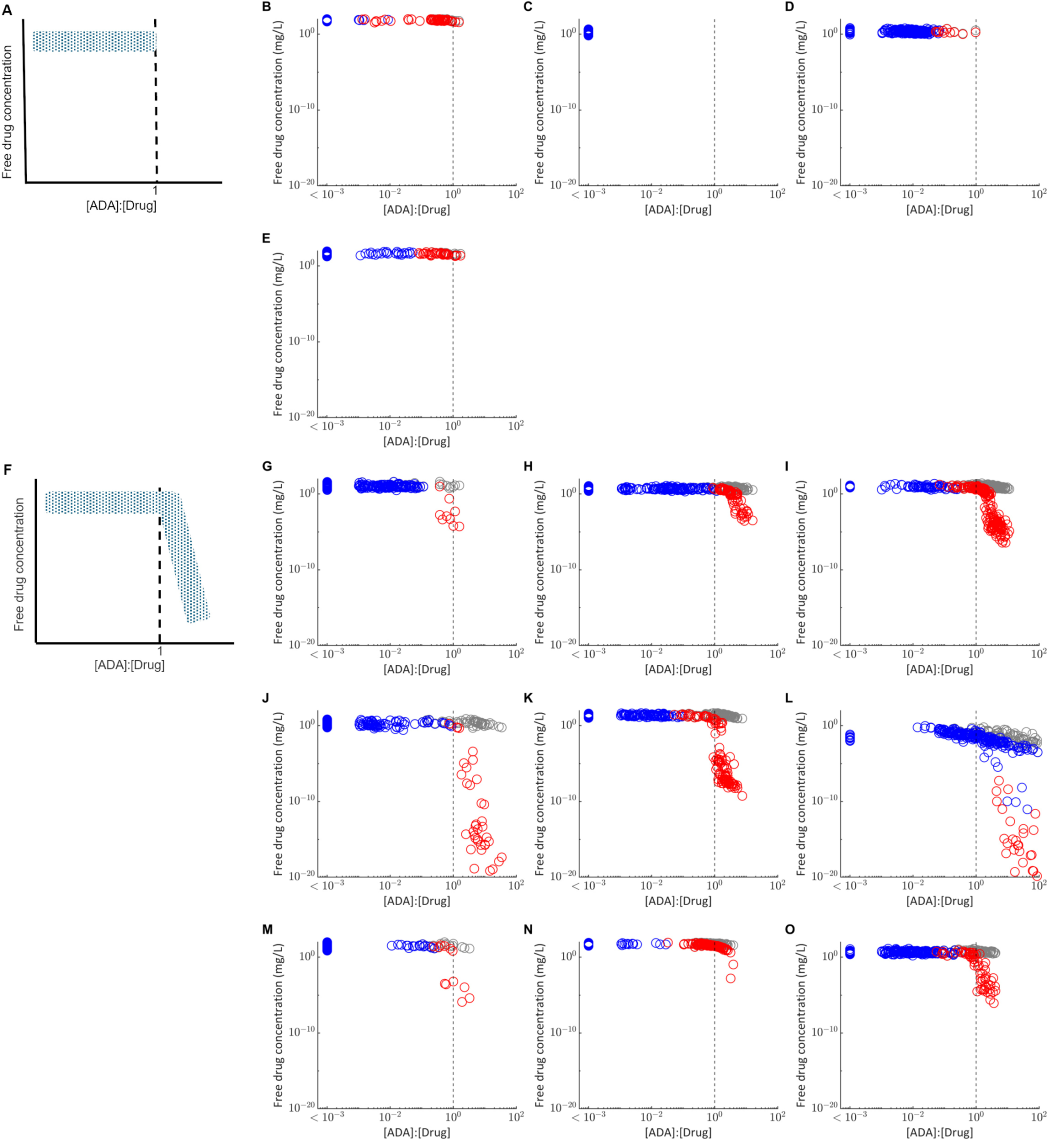
Relationship between the molar ratio of the ADA concentration to drug concentration ([ADA]:[Drug]) and the free drug concentration in plasma. Each point represents the free drug concentration and [ADA]:[Drug] at the final measurement time used in the matched clinical study design for virtual subjects classified as ADA positive (red) or ADA negative (blue). Grey points plot the free drug concentration in plasma for the same virtual subjects in the absence of ADA, simulated with the immune response model switched off. The grey dashed line indicates [ADA]:[Drug] equal to 1. **(A)** Fingerprint profile for drugs in which ADA have not impact on PK as observed for **(B)** bevacizumab **(C)** etanercept **(D)** ixekizumab and **(E)** tocilizumab. **(F)** Fingerprint profile for drugs in which ADA have a significant impact on PK, as observed for **(G)** adalimumab **(H)** bococizumab **(I)** certolizumab pegol **(J)** infliximab **(K)** natalizumab **(L)** rituximab **(M)** secukinumab **(N)** trastuzumab **(O)** ustekinumab. For visualization, all virtual subjects with [ADA]:[Drug] < 1E-3 are plotted as a single group.

To inform understanding of why ADA positivity was associated with a significant reduction in plasma concentration for some drugs but not others, the relationship between the predicted drug concentration and molar ratio of the ADA concentration versus drug concentration in the absence of ADA was assessed (**Figure 2**). Two fingerprint profiles were identified. For drugs where ADA does not significantly affect PK, the free drug concentration remains independent of the [ADA]:[Drug] ratio (**Figures 2A–E**). Furthermore, the [ADA]:[Drug] ratio is less than 1 for most virtual subjects and never exceeds 3. For drugs where ADA significantly impacts PK, free drug concentration is independent of [ADA]:[Drug] for a threshold of less than approximately 1, and drug concentrations are reduced when [ADA]:[Drug] exceeds this threshold (**Figure 2F–O**). The impact of [ADA]:[Drug] between 0.3 and 3 on PK is variable depending on both the compound and the individual, suggesting that variability in multiple PK and immune response parameters likely determine the exact impact of ADA on drug concentration. When the [ADA]:[Drug] is less than 0.3, minimal impact of ADA on drug concentration (<50% reduction) is consistently observed. However, when [ADA]:[Drug] exceeds 3, there is a pronounced reduction in free drug concentration to less than 90% of the predicted concentration in the absence of ADA.

For most of the drugs simulated, all virtual subjects for whom [ADA]:[Drug] exceeds 1 were classified as ADA positive, but not all individuals classified as ADA positive exceed this threshold. Rituximab is an exception (**Figure 2I**), with results suggesting there is an ADA dependent reduction in free drug concentration for some ADA negative individuals, likely due to the long time between last dose and the final plasma concentration measurement (22 weeks).

**Figure 3:**
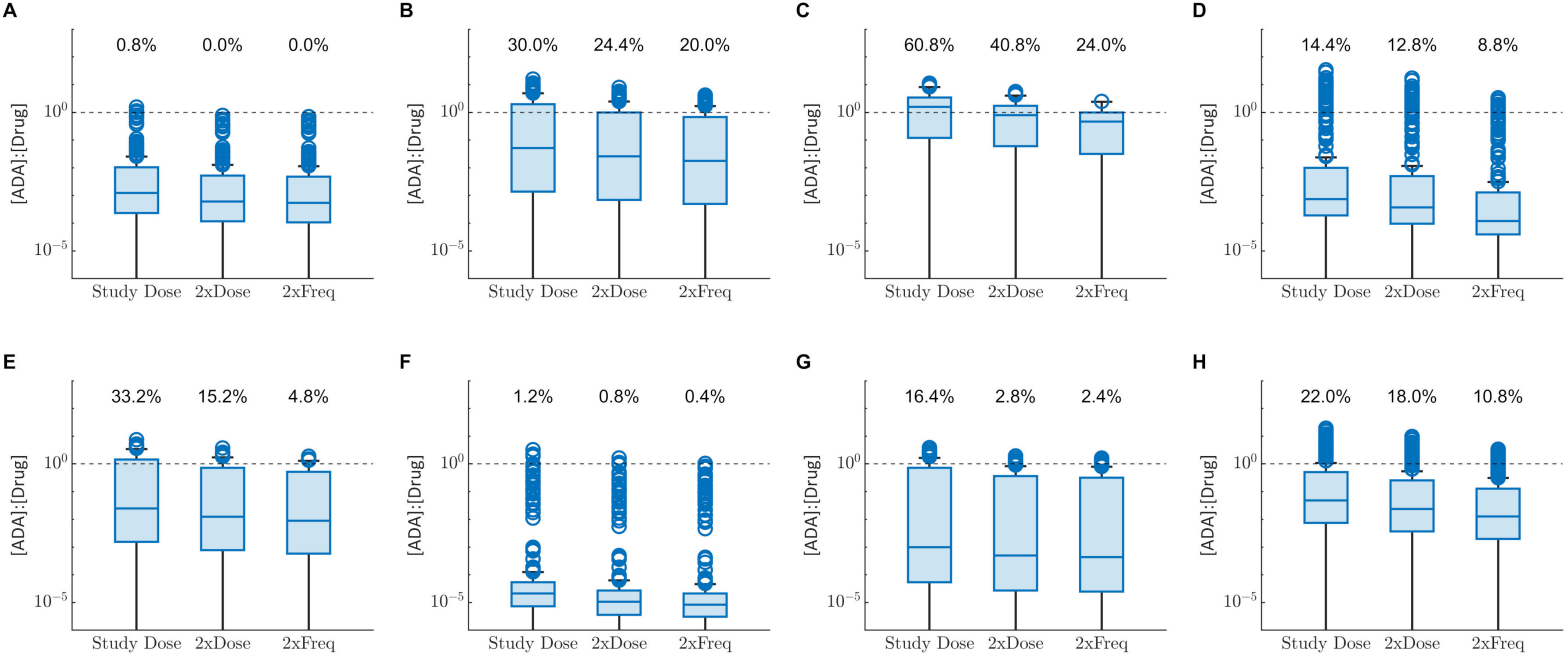
Box plots comparing the model predicted distribution of the [ADA]:[Drug] at C_trough_ for the clinical dosing regimen (Study Dose), twice the clinical dose amount (2xDose) or half the dosing interval (2xFreq). Percentages at the top of each plot display the percentage of virtual subjects with [ADA]:[Drug] >1. **(A)** Adalimumab, **(B)** bococizumab, **(C)** certolizumab pegol, **(D)** infliximab, **(E)** natalizumab, **(F)** secukinumab **(G)** trastuzumab, **(H)** ustekinumab. Points represent outliers that fall >1.5 times the interquartile range outside the 25^th^ and 75^th^ percentile and whiskers extend to the final non-outlier datapoint. Black dashed line corresponds to [ADA]:[Drug] equal to 1.

Since the impact of ADA on PK is dependent on drug concentration as well as ADA concentration, either increasing the dose amount or reducing the dosing interval may be viable approaches to manage the impact of ADA and maintain efficacious drug concentrations. We explored the impact of doubling the dose amount or halving the dosing interval on the number of individuals for which [ADA]:[Drug] exceeded 1 (**Figure 3**). For all drugs, both approaches resulted in a decrease in fraction of individuals for which [ADA]:[Drug] exceeds 1, suggesting the potential for ADA to be managed via dose adjustment in some individuals.

## 4 Discussion

Preclinical immunogenicity assessment typically relies on *in silico* and *in vitro* assessment methods that focus on single mechanisms and pathways associated with sequence-based immunogenicity risk. QSP modelling is a novel, evolving approach to immunogenicity assessment that integrates mathematical models capable of capturing the inherent complexity of the immune cell interactions with predictions from multiple immunogenicity risk assessment methods to predict risk of ADA development and the clinical relevance of ADA.

This manuscript evaluates results from application of a QSP model to predict both the incidence and clinical relevance of ADA development with respect to impact on drug PK for 13 monoclonal antibodies or Fc fusion proteins. The model accurately predicted whether there was a significant impact of ADA on PK for 10 out of 13 evaluated biotherapeutics. It successfully identified three cases where ADA positivity was not associated with a reduction in drug concentration for the reported clinical study design. In all seven instances where ADA positivity was reported to reduce plasma drug concentration, this outcome was predicted correctly. For two cases, ADA was incorrectly predicted to have an impact on PK. For the final case, etanercept, no subjects were predicted to be ADA positive so the predicted impact of ADA on PK could not be assessed. However, the conclusion from the model is that there is no formation of ADA or impact of immunogenicity on drug PK, in agreement with the low incidence of transient ADA that was not associated with any impact on drug PK reported in the clinical study.

Further analysis of the relationship between predicted drug and ADA concentrations revealed that both the drug and ADA concentrations determine whether there is an impact of ADA on PK. A reduction in drug concentration, measured at C_trough_ for most biotherapeutics evaluated, was typically only observed when molar ADA concentration exceeded the molar drug concentration. This is consistent both with observations from multiple clinical trials for example (30, 31), that higher ADA concentrations were associated with greater reduction in drug concentration, and clinical experience that increased dose amount or frequency can overcome loss of efficacy resulting from ADA for some drugs and patients. However, the impact of drug dose and frequency on [ADA]:[Drug] can be complex, as both parameters can influence ADA formation itself, which complicates interpretation.

We propose the [ADA]:[Drug] as a novel model-informed metric to quantify the risk of ADA impacting PK. This metric may be used to assess the potential to successfully manage clinical ADA by modifying the dosing during preclinical and early clinical development when understanding of the clinical concentration-response relationship is limited. Limits on feasible dose amount or frequency can be selected by considering drug solubility, toxicity and other available evidence, and used to explore the impact of dose on the proportion of virtual subjects for which [ADA]:[Drug] exceeds 1. In later phase clinical development, the QSP model can be calibrated to clinical ADA and PK data (32) extended with a pharmacodynamic model relating drug concentration to efficacy for a more thorough evaluation of the impact of ADA on efficacy. Furthermore, the importance of [ADA]:[Drug] suggests that for when a higher drug concentration is maintained throughout the dosing interval, the threshold ADA concentration that results in a reduction in drug concentration is also higher. This understanding also helps to inform understanding of the minimum ADA concentration sensitivity to detect ADA that have the potential to impact PK.

A limitation of the current work is that it has focused on a small test set of monoclonal antibodies and Fc fusion proteins with half-lives in the range of 4–28 days. Future work will analyse the applicability [ADA]:[Drug] and the prediction of clinical immunogenicity for a larger validation set of drugs. Validation with more diverse modalities such as bispecific antibodies, peptides, and endogenous proteins with a broader range of half-lives and therapeutic concentrations will enable better assessment of the generalisability of this metric to inform understanding of the risk of ADA impacting PK.

We were less successful in predicting the incidence of clinical immunogenicity, suggesting that challenges remain in the accurate prediction of the multiple biological mechanisms leading to immunogenicity. For this work, we integrated the results from experimental immunogenicity assessment commonly performed in industry, including dendritic cell uptake, the initial fraction of drug-specific naïve T cells, and MAPPs assays, with *in silico* prediction of HLA binding and clinical PK and study design data. However, there were gaps in the available data for our test set of drugs, and a lack of harmonisation assay protocols (33) may impact interpretation and translation of outcomes. While the small size of our test set precludes making definitive conclusions, we note a trend of reduced overprediction of immunogenicity for compounds in which more experimental data were available.

The current experimental and *in silico* approaches used to inform immunogenicity prediction focus on the role of dendritic cell and CD4^+^ T cell pathways, which do not fully capture the multifactorial nature of immune activation and regulation. To improve predictive accuracy, we suggest the continued development of experimental and *in silico* approaches to characterise additional mechanisms. Not all presented peptides activate CD4+ T cells, and improvements to *in silico* approaches for T cell receptor–peptide–MHCII interaction prediction will improve overprediction of potential T cell epitopes. Improved approaches to predict B cell activation may help to better characterise the contribution of B cells to immunogenicity (35). Understanding the immunogenic risk associated with drug-target interactions and drug mechanism of action may better inform predictions for individual drugs. For instance, the formation of immune complexes between adalimumab and its target, tumour necrosis factor alpha (TNFα), has been proposed to enhance the immunogenicity of adalimumab by promoting increased uptake by dendritic cells and subsequent antigen presentation (36). Furthermore, clinical observation of immunogenicity of nivolumab when co-administered with ipilimumab, suggest a role for T regulatory cells in modulating immune responses. Thus, developing predictive tools to assess drug-specific T regulatory interactions represents a promising avenue for enhancing immunogenicity risk assessment (32, 34).A further challenge is the high variability in the incidence of immunogenicity for the same drug across multiple clinical studies reported by this study and others (37). This makes it difficult to develop the QSP model since there is no single, fully quantitative and reproducible clinical data set for calibrating and validating model predictions. While the inconsistencies in the data likely in part reflects differences in study design (e.g. dosing regimen, co-medication) and study population (e.g. disease, ethnicity), the lack of standardisation of ADA assays has been identified as a major barrier to comparing results across clinical studies (37). Improved standardisation of ADA assays, development of bioanalysis methods to enable quantification of ADA concentrations and increased publication of ADA concentration data would better inform calibration and validation of QSP models for immunogenicity.

## 5 Conclusion

In conclusion, our findings highlight the potential of the QSP modelling approach to assess the risk of clinically relevant immunogenicity, particularly regarding the impact of ADA on drug PK. We introduce the [ADA]:[Drug] ratio as a model-informed metric to evaluate ADA effects on PK and to guide mitigation strategies, such as adjusting dose or frequency to sustain therapeutic drug levels. QSP models can integrate diverse non-clinical data across immunogenicity pathways. Developing these models alongside non-clinical assessment methods will improve immunogenicity prediction.

## Data Availability

The original contributions presented in the study are included in the article/**Supplementary Material**. Further inquiries can be directed to the corresponding author.
