# Supplementary material for: Towards new approach methodologies for biological therapeutics: a novel model-informed metric to assess immunogenicity risk
Source: Front Immunol. 2025 Nov 3;16:1677925. doi: 10.3389/fimmu.2025.1677925 (PMC12620829; doi:10.3389/fimmu.2025.1677925)
Supplement: Supplementary file 1 [file DataSheet1.docx]

Supplementary Material

# Supplementary Figures and Table

## Supplementary Figures


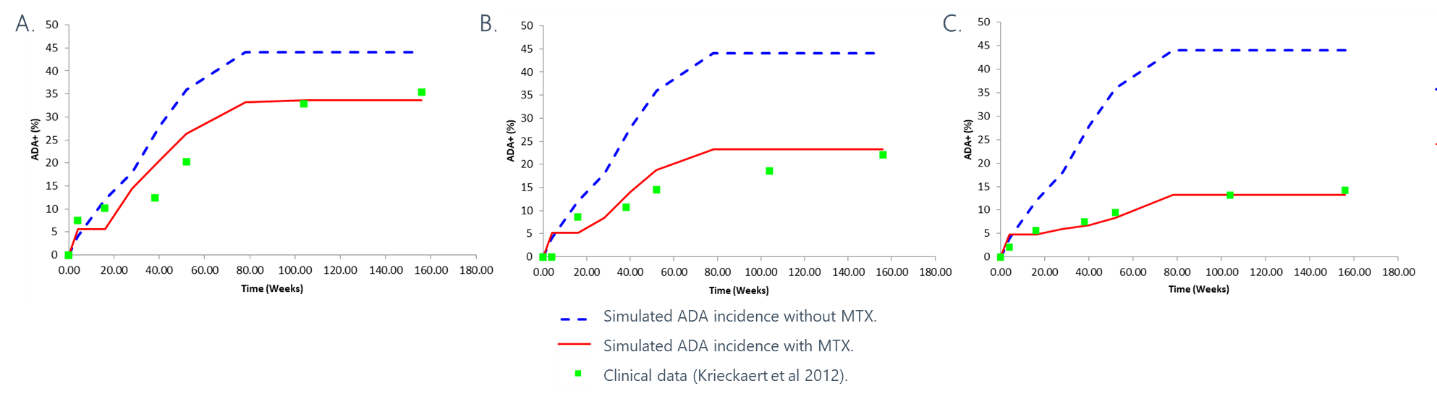


# Supplementary Figure 1. Predicted and observed impact of (A) low (5-10 mg/week), (B) intermediate (12.5-20 mg/week) or (C) high (≥ 22.5 mg/week) dose methotrexate on the ADA incidence for adalimumab.


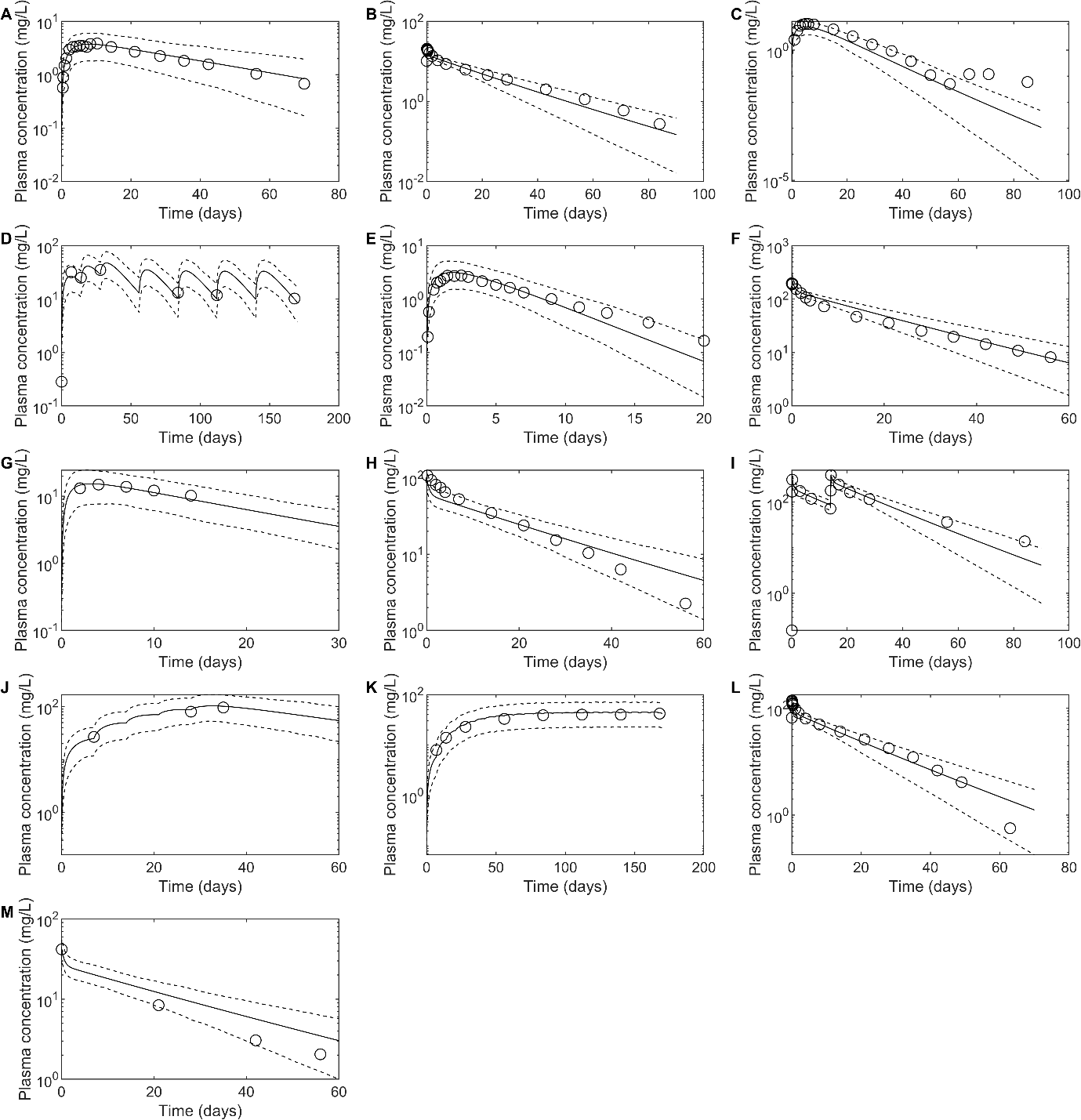


Supplementary Figure 2. Simulated (lines) and observed (open circles) plasma concentration profiles of biotherapeutics. Solid lines and points report the mean, dashed lines are the 5th and 95th percentiles for 250 simulated individuals. (A) Adalimumab 40 mg SC, single dose (1). (B) Bevacizumab 1 mg/kg IV, single dose (2). (C) Bococizumab 150 mg SC, single dose (3). (D) Certolizumab pegol 400 mg Q2W SC for three doses followed by nine 200mg Q2W doses (4). (E) Etanercept 50 mg subcutaneous, single dose (5). (F) Infliximab 10 mg/kg IV, single dose (6).(G) Ixekizumab 160 mg SC, single dose, (7). (H) Natalizumab 300 mg IV, single dose (8). (I) Rituximab 1000 mg SC administered on day 0 and day 14 (9). (J) Secukinumab 300 mg SC once weekly for 5 weeks (10). (K) Tocilizumab 162 mg SC (11). (L) Trastuzumab 6 mg/kg IV, single dose (12). (M) Ustekinumab 130 mg IV, single dose (13).

## Supplementary Tables

Supplementary Table 1. Compound-specific parameters describing the pharmacokinetics of test compounds.

| **Parameter** | **Molecular weight (g/mol)** | **fa** | **ka (1/h)** | **FcRn binding K_D_ pH6.0 (μM)** | **Systemic clearance (L/h)** |
| --- | --- | --- | --- | --- | --- |
| Adalimumab | 144190.3 (14) | 0.58 (30%) | 0.014 (30%) | 0.672 (10%) | 1 E-6 (10%) |
| Bevacizumab | 149000 (14) | n/a | n/a | 0.672 (10%) | 0.0061 (10%) |
| Bococizumab | 145100 (15) | 0.58 (30%) | 0.01 | 0.672 (10%) | 0.023 (10%) |
| Briakinumab | 147000 (14) | 0.58 (30%) | 0.01 (30%) | 0.672 (10%) | 0.024 (10%) |
| Certolizumab pegol | 91000 (14) | 0.58 (30%) | 0.01 (30%) | 0.672 (10%) | 0.01005 (10%) |
| Etanercept | 150000 (14) | 0.6 (30%) | 0.0257 (30%) | 3.612 (10%) (16) | 0.0345 (10%) |
| Infliximab | 144190.3 (14) | n/a | n/a | 0.672 (10%) | 0.006 (10%) |
| Ixekizumab | 146158 (14) | 0.6 (30%) | 0.03 (30%) | 0.672 (10%) | 0.007 (10%) |
| Natalizumab | 149000 (14) | n/a | n/a | 0.672 (10%) | 0.004 (30%) |
| Rituximab | 143859.7 (14) | n/a | n/a | 0.672 (10%) | 0.007 (10%) |
| Secukinumab | 151000 (14) | 0.58 (30%) | 0.001 (10%) | 0.672 (10%) | 0.001 (10%) |
| Tocilizumab | 148000 (14) | 0.355 (30%) | 0.0025 | 0.672 (10%) | 0.009 (10%) |
| Trastuzumab | 145531.5 (14) | n/a | n/a | 0.672 (10%) | 0.0081 (10%) |
| Ustekinumab | 148600 (14) | 0.58 (30%) | 0.01 (30%) | 0.672 (10%) | 0.0025 (10%) |

fa- fraction absorbed; ka – first order absorption rate; FcRn – neonatal Fc receptor; K_D_ – equilibrium dissociation rate.

n/a: Not applicable, IV administration.

Supplementary Table 2. Summary of experimental data used as inputs to the model.

| - **Compounds** | - **T cell epitope prediction method** | - **Ag Specific T Cells** - **(Cells per million)** | - **DC Internalisation Scalar (relative to bococizumab (mean, (CV)) (17)** | - **Reported ADA assay concentration threshold** |
| --- | --- | --- | --- | --- |
| - Adalimumab​ | - MAPPs (18, 19) | - 0.33 (20) | - 0.40 (58%) | - [ADA] >144 ng/mL, [ADA]high > 1200 ng/mL (21) |
| - Bevacizumab | - MAPPs (18) | - 0.2 (20) | - 0.055 (98%) | - [ADA] > 110 ng/mL |
| - Bococizumab | - MAPPs (18) | - NA | - 1 (36%) | - [ADA] > 250 ng/mL |
| - Certolizumab-Pegol | - Sequence | - NA | - NA | - NA |
| - Etanercept​ | - Sequence | - 0.01(20) | - 0.019 (135%) | - NA |
| - Infliximab​ | - MAPPs (18, 22) | - 0.1 (20) | - 0.050 (81%) | - NA |
| - Ixekizumab​ | - MAPPs (18, 23) | - 0.21 (23) | - 0.53 (76%) | - NA |
| - Natalizumab​ | - MAPPs (19) | - 2.2 (24) | - NA | - NA |
| - Rituximab​ | - MAPPs (22) | - 0.35 (20) | - NA | - [ADA] > 250 ng/mL |
| - Secukinumab​ | - MAPPs (23) | - 0.01 (25) | - 0.29 (71%) | - [ADA] > 250 ng/mL |
| - Tocilizumab | - Sequence | - 0.07 (26) | - NA | - [ADA] > 61.4 ng/mL |
| - Trastuzumab | - Sequence | - 0.02 (20) | - NA | - NA |
| - Ustekinumab | - Sequence | - 0.19 (25) | - NA | - NA |

MAPPs: MHC-associated peptide proteomics

NA: not available

Supplementary Table 3. Clinical trial design for comparator clinical studies.

| - **Compounds** | - **Dosing regimen** | - **Route of administration** | - **Study duration (weeks)** | - **Methotrexate comedication** | - **Reference** |
| --- | --- | --- | --- | --- | --- |
| - Adalimumab​ | - 40 mg Q2W | - SC | - 156 | - None | - (27) |
| - Bevacizumab | - 15 mg/kg Q3W | - IV | - 18 | - None | - (28) |
| - Bococizumab | - 150 mg Q2W | - SC | - 52 | - None | - (29) |
| - Certolizumab-Pegol | - 400 mg Q2W for 6 week induction phase then Q4W maintenance. | - SC | - 364 | - None | - (30) |
| - Etanercept​ | - 50 mg QW | - SC | - 52 | - 36% subjects co-administered immunosuppressants* | - (31) |
| - Infliximab​ | - 3 mg/kg Q2W for 2 doses then Q8W | - IV | - 244 | - None | - (32) |
| - Ixekizumab​ | - 160 mg dose then 80 mg Q2W for 12 weeks then 80 mg Q4W | - SC | - 60 | - 100% subjects co-administered methotrexate, 10-25 mg/week | - (33) |
| - Natalizumab​ | - 300 mg Q4W | - IV | - 120 | - 81% subjects co-administered methotrexate | - (34) |
| - Rituximab​ | - 2 x 1000 mg doses 2 weeks apart | - IV | - 24 | - None | - (35) |
| - Secukinumab​ | - 300 mg QW for 5 weeks then Q4W | - IV | - 52 | - None | - (36) |
| - Tocilizumab | - 162 mg QW | - SC | - 24 | - 100% subjects co-administered methotrexate. Average dose 15.5 mg/week | - (11) |
| - Trastuzumab | - 4 mg/kg induction dose then 2mg/kg QW | - IV | - 40 | - 50% subjects co-administered methotrexate, dose ≤ 25 mg/week. | - (37) |
| - Ustekinumab | - 130 mg induction dose then 90 mg Q8W | - IV induction, SC maintenance | - 52 | - 80% subjects co-administered methotrexate | - (13) |

IV – intravenous; SC – subcutaneous

QW – once weekly; QnW – once every n weeks.

*Immunosuppressants include methotrexate. Assumed that 25% of subjects received methotrexate.

# References

1. Hyland E, Mant T, Vlachos P, Attkins N, Ullmann M, Roy S, et al. Comparison of the pharmacokinetics, safety, and immunogenicity of MSB11022, a biosimilar of adalimumab, with Humira((R)) in healthy subjects. *Br J Clin Pharmacol* (2016) 82:983-93. doi: 10.1111/bcp.13039.

2. Wynne C, Schwabe C, Batra SS, Lopez-Lazaro L, Kankanwadi S. A comparative pharmacokinetic study of DRL_BZ, a candidate biosimilar of bevacizumab, with Avastin(®) (EU and US) in healthy male subjects. *Br J Clin Pharmacol* (2018) 84:2352-64. doi: 10.1111/bcp.13691.

3. Wang EQ, Plotka A, Salageanu J, Baltrukonis D, Mridha K, Frederich R, et al. Comparative Pharmacokinetics and Pharmacodynamics of Bococizumab Following a Single Subcutaneous Injection Using Drug Substance Manufactured at Two Sites or Administration via Two Different Devices. *Clin Pharmacol Drug Dev* (2019) 8:40-8. doi: 10.1002/cpdd.454.

4. European Medicines Agency. Assessment report: Cimzia. Procedure no. EMEA/H/C/001037/II/29 (2013). <https://www.ema.europa.eu/en/documents/variation-report/cimzia-h-c-1037-ii-0029-epar-assessment-report-variation_en.pdf>. [Accessed December, 2020].

5. Shennak M, Al-Jaouni R, Kshirasagar S, Kasibhatta RS, Godse N, Al-Ghazawi A, et al. An Open-Label, Randomized, Single-Dose, Crossover, Comparative Pharmacokinetics Study of YLB113 and the Etanercept Reference Product in Healthy Adult Male Subjects. *Eur J Drug Metab Pharmacokinet* (2020) 45:467-75. doi: 10.1007/s13318-020-00613-9.

6. FDA. APPLICATION NUMBER: 761072Orig1s000. CLINICAL PHARMACOLOGY AND BIOPHARMACEUTICS REVIEW(S) (2019). <https://www.accessdata.fda.gov/drugsatfda_docs/nda/2017/761072Orig1s000ClinPharmR.pdf>. [Accessed July 23, 2025].

7. Callis Duffin K, Bagel J, Bukhalo M, Mercado Clement IJ, Choi SL, Zhao F, et al. Phase 3, open-label, randomized study of the pharmacokinetics, efficacy and safety of ixekizumab following subcutaneous administration using a prefilled syringe or an autoinjector in patients with moderate-to-severe plaque psoriasis (UNCOVER-A). *J Eur Acad Dermatol Venereol* (2017) 31:107-13. doi: 10.1111/jdv.13768.

8. Plavina T, Fox EJ, Lucas N, Muralidharan KK, Mikol D. A Randomized Trial Evaluating Various Administration Routes of Natalizumab in Multiple Sclerosis. *J Clin Pharmacol* (2016) 56:1254-62. doi: 10.1002/jcph.707.

9. Cohen S, Emery P, Greenwald M, Yin D, Becker JC, Melia LA, et al. A phase I pharmacokinetics trial comparing PF-05280586 (a potential biosimilar) and rituximab in patients with active rheumatoid arthritis. *Br J Clin Pharmacol* (2016) 82:129-38. doi: 10.1111/bcp.12916.

10. Bruin G, Hasselberg A, Koroleva I, Milojevic J, Calonder C, Soon R, et al. Secukinumab Treatment Does Not Alter the Pharmacokinetics of the Cytochrome P450 3A4 Substrate Midazolam in Patients With Moderate to Severe Psoriasis. *Clin Pharmacol Ther* (2019) 106:1380-8. doi: 10.1002/cpt.1558.

11. Burmester GR, Rubbert-Roth A, Cantagrel A, Hall S, Leszczynski P, Feldman D, et al. A randomised, double-blind, parallel-group study of the safety and efficacy of subcutaneous tocilizumab versus intravenous tocilizumab in combination with traditional disease-modifying antirheumatic drugs in patients with moderate to severe rheumatoid arthritis (SUMMACTA study). *Ann Rheum Dis* (2014) 73:69-74. doi: 10.1136/annrheumdis-2013-203523.

12. Hanes V, Chow V, Zhang N, Markus R. A randomized, single-blind, single-dose study evaluating the pharmacokinetic equivalence of proposed biosimilar ABP 980 and trastuzumab in healthy male subjects. *Cancer Chemother Pharmacol* (2017) 79:881-8. doi: 10.1007/s00280-017-3286-9.

13. Adedokun OJ, Xu Z, Gasink C, Jacobstein D, Szapary P, Johanns J, et al. Pharmacokinetics and Exposure Response Relationships of Ustekinumab in Patients With Crohn's Disease. *Gastroenterology* (2018) 154:1660-71. doi: 10.1053/j.gastro.2018.01.043.

14. Knox C, Wilson M, Klinger CM, Franklin M, Oler E, Wilson A, et al. DrugBank 6.0: the DrugBank Knowledgebase for 2024. *Nucleic Acids Res* (2024) 52:D1265-D75. doi: 10.1093/nar/gkad976.

15. Wikipedia. Bococizumab (2023). <https://en.wikipedia.org/wiki/Bococizumab>. [updated December 1, 2023; Accessed July 30, 2025].

16. Suzuki T, Ishii-Watabe A, Tada M, Kobayashi T, Kanayasu-Toyoda T, Kawanishi T, et al. Importance of neonatal FcR in regulating the serum half-life of therapeutic proteins containing the Fc domain of human IgG1: a comparative study of the affinity of monoclonal antibodies and Fc-fusion proteins to human neonatal FcR. *J Immunol* (2010) 184:1968-76. doi: 10.4049/jimmunol.0903296.

17. Melendez R, Ordonia B, Guerrero J, Hassanzadeh A, Tran P, Low J, et al. Introducing dendritic cell antibody internalization as an immunogenicity risk assessment tool. *Bioanalysis* (2022) 14:703-13. doi: 10.4155/bio-2022-0024.

18. Hartman K, Steiner G, Siegel M, Looney CM, Hickling TP, Bray-French K, et al. Expanding the MAPPs Assay to Accommodate MHC-II Pan Receptors for Improved Predictability of Potential T Cell Epitopes. *Biology (Basel)* (2023) 12:1265. doi: 10.3390/biology12091265.

19. Meunier S, Hamze M, Karle A, de Bourayne M, Gdoura A, Spindeldreher S, et al. Impact of human sequences in variable domains of therapeutic antibodies on the location of CD4 T-cell epitopes. *Cell Mol Immunol* (2020) 17:656-8. doi: 10.1038/s41423-019-0304-3.

20. Delluc S, Ravot G, Maillere B. Quantitative analysis of the CD4 T-cell repertoire specific to therapeutic antibodies in healthy donors. *FASEB J* (2011) 25:2040-8. doi: 10.1096/fj.10-173872.

21. Bartelds GM, Krieckaert CL, Nurmohamed MT, van Schouwenburg PA, Lems WF, Twisk JW, et al. Development of antidrug antibodies against adalimumab and association with disease activity and treatment failure during long-term follow-up. *JAMA* (2011) 305:1460-8. doi: 10.1001/jama.2011.406.

22. Hamze M, Meunier S, Karle A, Gdoura A, Goudet A, Szely N, et al. Characterization of CD4 T Cell Epitopes of Infliximab and Rituximab Identified from Healthy Donors. *Front Immunol* (2017) 8:500. doi: 10.3389/fimmu.2017.00500.

23. Spindeldreher S, Karle A, Correia E, Tenon M, Gottlieb S, Huber T, et al. T cell epitope mapping of secukinumab and ixekizumab in healthy donors. *MAbs* (2020) 12:1707418. doi: 10.1080/19420862.2019.1707418.

24. Schultz HS, Reedtz-Runge SL, Backstrom BT, Lamberth K, Pedersen CR, Kvarnhammar AM, et al. Quantitative analysis of the CD4+ T cell response to therapeutic antibodies in healthy donors using a novel T cell:PBMC assay. *PLoS One* (2017) 12:e0178544. doi: 10.1371/journal.pone.0178544.

25. Spindeldreher S, Maillere B, Correia E, Tenon M, Karle A, Jarvis P, et al. Secukinumab Demonstrates Significantly Lower Immunogenicity Potential Compared to Ixekizumab. *Dermatol Ther (Heidelb)* (2018) 8:57-68. doi: 10.1007/s13555-018-0220-y.

26. Sigaux J, Hamze M, Daien C, Morel J, Krzysiek R, Pallardy M, et al. Immunogenicity of tocilizumab in patients with rheumatoid arthritis. *Joint Bone Spine* (2017) 84:39-45. doi: 10.1016/j.jbspin.2016.04.013.

27. Krieckaert CL, Nurmohamed MT, Wolbink GJ. Methotrexate reduces immunogenicity in adalimumab treated rheumatoid arthritis patients in a dose dependent manner. *Ann Rheum Dis* (2012) 71:1914-5. doi: 10.1136/annrheumdis-2012-201544.

28. Thatcher N, Goldschmidt JH, Thomas M, Schenker M, Pan Z, Paz-Ares Rodriguez L, et al. Efficacy and Safety of the Biosimilar ABP 215 Compared with Bevacizumab in Patients with Advanced Nonsquamous Non-small Cell Lung Cancer (MAPLE): A Randomized, Double-blind, Phase III Study. *Clin Cancer Res* (2019) 25:2088-95. doi: 10.1158/1078-0432.Ccr-18-2702.

29. Ridker PM, Tardif JC, Amarenco P, Duggan W, Glynn RJ, Jukema JW, et al. Lipid-Reduction Variability and Antidrug-Antibody Formation with Bococizumab. *N Engl J Med* (2017) 376:1517-26. doi: 10.1056/NEJMoa1614062.

30. Sandborn WJ, Wolf DC, Kosutic G, Parker G, Schreiber S, Lee SD, et al. Effects of Transient and Persistent Anti-drug Antibodies to Certolizumab Pegol: Longitudinal Data from a 7-Year Study in Crohn's Disease. *Inflamm Bowel Dis* (2017) 23:1047-56. doi: 10.1097/MIB.0000000000001100.

31. Emery P, Vencovský J, Sylwestrzak A, Leszczyński P, Porawska W, Baranauskaite A, et al. A phase III randomised, double-blind, parallel-group study comparing SB4 with etanercept reference product in patients with active rheumatoid arthritis despite methotrexate therapy. *Ann Rheum Dis* (2017) 76:51-7. doi: 10.1136/annrheumdis-2015-207588.

32. Pascual-Salcedo D, Plasencia C, Ramiro S, Nuño L, Bonilla G, Nagore D, et al. Influence of immunogenicity on the efficacy of long-term treatment with infliximab in rheumatoid arthritis. *Rheumatology (Oxford)* (2011) 50:1445-52. doi: 10.1093/rheumatology/ker124.

33. Reich K, Jackson K, Ball S, Garces S, Kerr L, Chua L, et al. Ixekizumab Pharmacokinetics, Anti-Drug Antibodies, and Efficacy through 60 Weeks of Treatment of Moderate to Severe Plaque Psoriasis. *J Invest Dermatol* (2018) 138:2168-73. doi: 10.1016/j.jid.2018.04.019.

34. Calabresi PA, Giovannoni G, Confavreux C, Galetta SL, Havrdova E, Hutchinson M, et al. The incidence and significance of anti-natalizumab antibodies: results from AFFIRM and SENTINEL. *Neurology* (2007) 69:1391-403. doi: 10.1212/01.wnl.0000277457.17420.b5.

35. Park W, Božić-Majstorović L, Milakovic D, Berrocal Kasay A, El-Khouri EC, Irazoque-Palazuelos F, et al. Comparison of biosimilar CT-P10 and innovator rituximab in patients with rheumatoid arthritis: a randomized controlled Phase 3 trial. *MAbs* (2018) 10:934-43. doi: 10.1080/19420862.2018.1487912.

36. Deodhar A, Gladman DD, McInnes IB, Spindeldreher S, Martin R, Pricop L, et al. Secukinumab Immunogenicity over 52 Weeks in Patients with Psoriatic Arthritis and Ankylosing Spondylitis. *J Rheumatol* (2020) 47:539-47. doi: 10.3899/jrheum.190116.

37. Pegram MD, Bondarenko I, Zorzetto MMC, Hingmire S, Iwase H, Krivorotko PV, et al. PF-05280014 (a trastuzumab biosimilar) plus paclitaxel compared with reference trastuzumab plus paclitaxel for HER2-positive metastatic breast cancer: a randomised, double-blind study. *Br J Cancer* (2019) 120:172-82. doi: 10.1038/s41416-018-0340-2.

# 
